# Flexible Polypyrrole-Based Composite Films with Tailored Electrical and Mechanical Properties for Electrocardiographic Sensing

**DOI:** 10.3390/polym18060779

**Published:** 2026-03-23

**Authors:** Alin-Alexandru Andrei, Izabell Craciunescu, Lucian Barbu Tudoran, Rodica Paula Turcu, George Marian Ispas, Gavril-Ionel Giurgi, Alexandru Oprea, Mioara Zagrai, Cristian Sevcencu

**Affiliations:** 1 National Institute for Research and Development of Isotopic and Molecular Technologies, 400293 Cluj-Napoca, Romania; alin.andrei@itim-cj.ro (A.-A.A.); lucian.barbu@ubbcluj.ro (L.B.T.); rodica.turcu@itim-cj.ro (R.P.T.); george.ispas@itim-cj.ro (G.M.I.); gavril.giurgi@itim-cj.ro (G.-I.G.); alexandru.oprea@itim-cj.ro (A.O.); mioara.zagrai@itim-cj.ro (M.Z.); cristian.sevcencu@itim-cj.ro (C.S.); 2 Doctoral School in Integrative Biology, Faculty of Biology and Geology, Babes-Bolyai University, 400084 Cluj-Napoca, Romania

**Keywords:** polypyrrole, conductive polymer composites, flexible electrodes, electrical and mechanical properties, electrocardiographic sensing, bioelectronic materials

## Abstract

Flexible electrode materials with tailored electrical and mechanical properties are essential for reliable electrocardiographic (ECG) sensing. In this work, p-toluenesulfonic-acid-doped polypyrrole (PPy–TSA) films were modified using polymeric and inorganic fillers, as well as their combinations (polyethylene glycol, graphene, carbon nanotubes, and zeolite), to tune their functional performance. The reference PPy–TSA film exhibits typical morphological and chemical characteristics of doped polypyrrole and serves as a reliable baseline for comparison. All composite films retain electrical conductivity within the range required for ECG applications while showing improved mechanical compliance (i.e., enhanced ability to conform to the skin and sustain deformation). Based on the optimized balance between electrical and mechanical properties, flexible ECG electrodes were fabricated using the TSA-doped PPy-based composite film. ECG recordings obtained with the several proposed electrodes show good agreement with those acquired using a commercial ECG electrode, demonstrating the potential of PPy-based composite films for flexible bioelectronic sensing applications.

## 1. Introduction

Polypyrrole (PPy) is an intrinsically conducting polymer that has been extensively explored in sensor technologies, bioelectronic and biomedical applications, and wearable devices, owing to its electrical conductivity, ease of synthesis, and favorable electrochemical behavior [1,2,3]. Due to these characteristics, PPy-based films and coatings have been explored for applications such as physiological signal monitoring, including electrocardiographic (ECG) sensing, where a stable electrical contact and compatibility with flexible substrates are required [4,5].

Recent advances in advanced functional materials have emphasized the importance of compositional engineering and structure–property relationships in optimizing performance across a wide range of applications [6,7].

However, the use of pure PPy is accompanied by several limitations that restrict its direct implementation in wearable and skin-contact applications [8,9]. Pure PPy films are typically fragile and show limited mechanical flexibility as well as poor stability under repeated deformation [10,11]. Such mechanical limitations can lead to cracking and loss of electrical contact during dynamic operation. In addition, the relatively high stiffness of PPy may reduce user comfort when devices are intended for prolonged contact with the skin. These aspects indicate the need for material strategies that preserve the electrical functionality of PPy while improving its mechanical stability and suitability for wearable bioelectronic applications.

To overcome the intrinsic mechanical limitations of pure PPy, various material strategies have been explored, most commonly based on blending PPy with other polymers or incorporating functional fillers to form composite systems [12,13]. The introduction of elastomeric or flexible polymer matrices, as well as conductive nanomaterials such as carbon nanotubes or graphene derivatives, has been reported to improve mechanical flexibility and resistance to deformation [14,15,16,17]. However, such modifications could also influence the electrical, structural, or optical properties of PPy, and in some cases could lead to reduced electrical conductivity or increased material heterogeneity. Therefore, achieving an appropriate balance between mechanical stability and functional electrical performance remains a key challenge, particularly for applications requiring reliable biological signal acquisition under dynamic conditions, such as wearable bioelectronic sensors.

In this work, a comprehensive characterization of PPy-based composite films was performed, aiming to address the mechanical limitations of pure PPy while preserving its key functional properties. The investigated systems include doped PPy-based composites incorporating, on the one hand, polymeric materials such as poly (vinyl alcohol) (PVA) and poly (ethylene glycol) (PEG), and, on the other hand, conductive and inorganic fillers, namely carbon nanotubes, graphene, and zeolite. The polymeric additives, PVA and PEG, were selected to introduce softer and more compliant domains within the PPy matrix, thereby promoting increased flexibility and a higher elasticity. Previous studies have reported the incorporation of polymeric additives such as PVA or PEG into PPy-based systems as an effective strategy to improve mechanical compliance, flexibility, and overall processability of the resulting materials [18,19,20,21]. However, due to the electrically insulating nature of these polymers, their addition has often been associated with a decrease in electrical conductivity when used in higher amounts. This observation highlights the need for a carefully balanced additive content, an aspect that remains insufficiently explored in relation to the simultaneous preservation of electrical and sensing-related properties.

In line with previous reports [22,23,24,25,26], carbon nanotubes and graphene were employed in this study as reinforcing conductive fillers to enhance the mechanical strength and structural integrity of the material while maintaining its efficient charge transport. Nevertheless, the incorporation of such rigid nanofillers has been shown to increase the stiffness of PPy-based composites, often resulting in mechanically robust but less compliant films, which may be less suitable for applications requiring soft and skin-conformable materials.

Zeolite was introduced as an inorganic component to generate additional porosity within the composite films, in line with previous studies on PPy–zeolite composites. The porous nature of zeolite can influence both the microstructural organization and adsorption capability of the material, potentially enabling additional functionalities in future bioelectronic applications. Notably, zeolite-containing composites have been previously investigated in gas sensing and chemical detection applications, where the adsorption of target species within the microporous zeolite framework plays a key functional role [27,28,29].

Building on the additive strategies reported in the literature, this study further explored a representative hybrid composite formulation combining polymeric components with inorganic fillers. This approach was deliberately adopted to balance elastic compliance and mechanical reinforcement within a single material system and so addresses limitations commonly encountered when these strategies are applied independently. The synthesis protocols were designed to avoid significant structural modifications of the PPy matrix that could affect its electrical and optical properties. Instead, the selected additives were introduced in a controlled manner, with the objective of improving the mechanical behavior of the resulting films, particularly in terms of flexibility and stability under deformation. A comprehensive structural, optical, electrical, and mechanical evaluation was conducted to assess the influence of each additive on the overall material performance.

The study was conducted from a validation-oriented perspective, using a representative sensing application, namely ECG electrodes, in order to investigate if the proposed material modifications lead to improved mechanical properties of such electrodes without adversely affecting other characteristics relevant to the sensing process, including their electrical and optical performance. All investigated materials were subsequently integrated into ECG electrode configuration and evaluated through direct ECG signal acquisition, with the recorded signals compared to those obtained using a commercial ECG electrode.

## 2. Materials and Methods

### 2.1. Materials

Pyrrole (Py), ≥98%; p-toluenesulfonic acid (p-TSA), ≥98%; and acetonitrile (ACN), HPLC grade, were purchased from Sigma-Aldrich and used as received. Poly (vinyl alcohol) (PVA, Mw ≈ 120,000) and poly (ethylene glycol) (PEG, Mw ≈ 10,000) were also obtained from Sigma-Aldrich and used as received. Graphene and carbon nanotubes were synthesized locally at the institute using established in-house procedures. Natural zeolite, extracted from the Rupea area (Brașov County, Romania), was kindly provided by Zeolite Developments.

### 2.2. Synthesis of Doped PPy-Based Composite Films

Polypyrrole (PPy) films were synthesized by electrochemical polymerization using a galvanostatic deposition approach at constant current, controlled by a Voltcraft DSP-6010 (Conrad Electronic, Hirschau, Germany) power supply. p-Toluenesulfonic acid (p-TSA) was used as a dopant due to its strong acidic character and bulky aromatic structure, which facilitate efficient doping of polypyrrole, improve charge delocalization, and enhance both electrical conductivity and film-forming properties. The polymerization was carried out in a three-electrode electrochemical cell using a working electrode and a counter electrode made of stainless steel (surface area 4 × 4 cm^2^), and an Ag/AgCl reference electrode was cleaned by ultrasonication in ethanol and thoroughly rinsed with distilled water.

The electrochemical polymerization was performed in 120 mL of ACN as polymerization medium containing p-TSA, (0.1 M, 2 g) as dopant and Py monomer (0.1 M, 0.85 mL). The deposition was conducted in galvanostatic conditions at a constant current of I = 0.032 A for a polymerization time of 1 h. After polymerization, the resulting PPy films were rinsed successively with ethanol and distilled water to remove residual electrolyte and loosely bound species, and subsequently detached from the stainless-steel substrate to obtain free-standing films.

For the preparation of PPy-based composite films, selected additives were introduced into the polymerization medium prior to electrochemical deposition. Pyrrole and p-toluenesulfonic acid (p-TSA) were used as monomer and dopant, respectively, both at a concentration of approximately 0.1 M in the aqueous polymerization solution.

Polyethylene glycol (PEG) was added in an amount corresponding to 18 wt% relative to the total solid content (0.62 g), while poly(vinyl alcohol) (PVA) was incorporated at a concentration of 13 wt% (0.432 g). Graphene (GR) and carbon nanotubes (CNT) were introduced as conductive fillers in an amount of 0.012 g, corresponding to approximately 0.4 wt% relative to the total solid mass. Zeolite (ZE) was added in an amount of 0.12 g, corresponding to approximately 4 wt% relative to the total solid mass. The concentrations of all additives were selected based on preliminary optimization experiments and literature data, aiming to achieve a balance between electrical conductivity, mechanical properties, and film-forming ability. The different loadings reflect the distinct roles of the additives (conductive versus structural modifiers) and were chosen to ensure efficient performance without compromising the integrity of the composite films.

In all cases, the additives were first dissolved or dispersed in the reaction medium under continuous stirring and then introduced into the polymerization solution prior to electrochemical deposition. To improve the homogeneity of the reaction medium, magnetic stirring was maintained throughout the entire polymerization process at a constant speed of 300 rpm. In addition to the individual additive systems, a representative hybrid composite combining both PEG and inorganic filler was synthesized using the same deposition parameters, in order to evaluate the combined effect of polymeric and inorganic components on the mechanical and functional properties of the resulting films.

All synthesis parameters and experimental conditions used for the preparation of the reference PPy sample and PPy-based composite films are systematically summarized in Table 1. Several preliminary batches were prepared in order to optimize the synthesis parameters. After establishing the final preparation conditions, the reproducibility of the method was verified by repeating the synthesis under identical conditions three times for each composition. The obtained films showed consistent morphological, mechanical, and electrical properties, confirming the reliability of the experimental procedure.

### 2.3. Characterization Methods

The morphology of the magnetic composite materials was investigated by Scanning Electron Microscopy (SEM) measurements (Hitachi HD-2700, Hitachi High-Technologies Corporation, Tokyo, Japan). X-ray Photoelectron Spectroscopy (XPS) measurements were made using an XPS spectrometer SPECS (SPECS Surface Nano Analysis GmbH, Berlin, Germany) equipped with a dual-anode X-ray source Al/Mg. The mechanical properties of the PPy-based films were investigated by dynamic mechanical analysis (DMA) using a DMA7100 instrument (Hitachi, Tokyo, Japan). The electrical properties of the prepared polypyrrole-based films were evaluated using the four-point probe method. Measurements were carried out using a custom-built setup consisting of a controlled current source and a voltage measurement unit. The sheet resistance was obtained from current–voltage data, and the electrical conductivity (σ) was calculated by considering the sample geometry, including film thickness and probe spacing. UV–Vis diffuse reflectance spectroscopy (UV–Vis DRS) was carried out using Perkin-Elmer Lambda 45 UV/Vis spectrometer (PerkinElmer Inc., Waltham, MA, USA). with a resolution of 0.1 nm. Absorption spectra were recorded over the 300–850 nm wavelength range.

### 2.4. ECG Signal Acquisition Using PPy-Based Film Electrodes

ECG signals were simultaneously recorded with common ECG electrodes and electrodes made from the tested PPy-based films. The seven PPy electrodes were made from 1 mm/1 mm squares of PPy films to which the leads conducting the ECG signals to the amplifier were connected with silver epoxy (Agar Scientific Ltd., Stansted, UK). Those PPy electrodes were integrated into custom-designed patch electrodes, which were made from common 5 cm diameter ECG adhesive pad electrodes having a classic Ag/AgCl contact in the middle and the seven PPy electrodes attached around, on the same surface with, and at an equal distance from the Ag/AgCl contact. Three such 8-contact patch electrodes were used to simultaneously record 3-lead ECGs through eight recording channels, i.e., one channel comprising 1 reference and 2 active Ag/AgCl contacts, and the other seven comprising the tested PPy contacts assembled in the same configuration.

The patch electrodes described above were placed on the chest of a human subject according to the standard ECG configuration. The ECG signals recorded through all eight channels were treated exactly the same, i.e., amplified 1000 times using an Iso-DAM 8A amplifier (World Precision Instruments, Sarasota, FL, USA), low pass (0.1 Hz) filtered and fed to a computer through a Power 1401 data acquisition system (Cambridge Electronic Design Ltd., Cambridge, UK). operated by Spike 2 software (Cambridge Electronic Design Ltd., Cambridge, UK). The ECG recordings were performed under resting conditions.

## 3. Results and Discussion

To evaluate the outcome of the electrochemical deposition process and to assess the influence of different additives on the properties of the PPy-based films, a comprehensive set of characterization techniques was employed. These measurements were carried out to highlight the effects of the incorporated additives on the morphological, structural, mechanical, electrical, and optical properties of the films, as well as to identify the most suitable material composition for the targeted application.

### 3.1. Morphological Properties of PPy-Based Films

**Figure 1:**
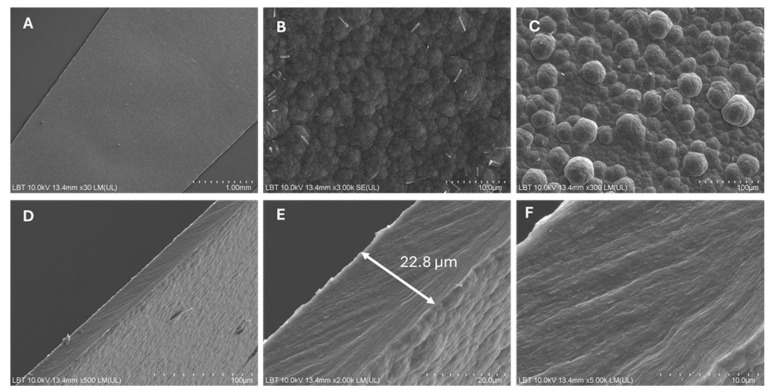
SEM images of the doped PPy–TSA reference film at different magnifications: (**A**–**C**) surface morphology and (**D**–**F**) cross-sectional views.

The surface morphology of the doped PPy and PPy-based composite films was investigated by scanning electron microscopy (SEM). The free-standing films were mounted on conductive sample holders and, when necessary, coated with a thin conductive layer in order to minimize charging effects during observation. SEM images were acquired at different magnifications to evaluate surface features, homogeneity, and the influence of the incorporated additives on film morphology. The surface morphology of the doped PPy–TSA reference film was investigated by SEM at different magnifications, as shown in Figure 1.

At low magnification (Figure 1A), the film surface appears continuous and relatively smooth, with no visible cracks, pinholes, or macroscopic defects, indicating a uniform deposition during the galvanostatic electropolymerization process. Upon increasing the magnification (Figure 1B), the surface reveals a more textured morphology, with the emergence of characteristic granular features. At high magnification (Figure 1C), the typical cauliflower-like morphology of polypyrrole films becomes clearly evident, consisting of densely packed globular structures with relatively uniform size distribution. This morphology is commonly associated with electrochemically synthesized PPy and is attributed to the nucleation-and-growth mechanism during polymerization under constant current conditions, being in good agreement with previous reports on p-TSA-doped polypyrrole films [30,31,32,33].

Cross-sectional SEM images (Figure 1D–F) further confirm the compact and homogeneous nature of the film, revealing a layered internal structure without apparent delamination or large voids. The absence of significant structural defects across the film thickness suggests good mechanical integrity and adhesion between the growing PPy layers during electrochemical deposition.

Based on these cross-sectional SEM images, the film thickness was determined by measuring the distance between the substrate and the film surface at multiple positions along the cross section, and the resulting values, expressed as mean thickness ± standard deviation, are reported in Table 1.

**Figure 2:**
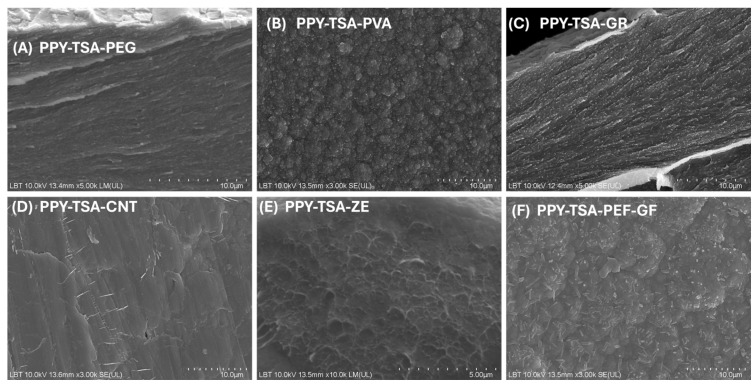
SEM images of doped PPy-based composite films containing different additives: (**A**) PPy–TSA–PEG, (**B**) PPy–TSA–PVA, (**C**) PPy–TSA–GR, (**D**) PPy–TSA–CNT, (**E**) PPy–TSA–Zeolite, and (**F**) PPy–TSA–PEG–GR.

SEM images illustrating the surface morphology of doped PPy-based composite films containing various polymeric and inorganic additives, recorded at the same magnification for direct comparison, are presented in Figure 2.

The incorporation of polymeric and inorganic additives leads to noticeable modifications of the surface morphology compared to the doped PPy–TSA reference film. The PPy–TSA–PEG composite film (Figure 2A) exhibits a more compact and layered surface morphology, with a reduced degree of surface roughness. This behavior can be attributed to the presence of PEG, which likely acts as a soft template during electropolymerization, promoting a more uniform growth of the PPy matrix. In the case of PPy–TSA–PVA (Figure 2B), the characteristic granular morphology of PPy remains clearly visible, although a more homogeneous distribution of globular features can be observed. The presence of PVA appears to influence the nucleation process, leading to a finer and more evenly distributed surface texture.

The addition of graphene (PPy–TSA–GR, Figure 2C) results in a surface morphology characterized by elongated and layered features, indicating the incorporation of graphene within the PPy matrix. Although graphene is present in the form of nanosheets, their lateral dimensions and orientation within the polymeric phase led to an acicular-like appearance in the SEM images at the selected magnification. The graphene nanosheets appear uniformly distributed throughout the polymer matrix, without the formation of large agglomerates, suggesting good dispersion and favorable interaction with the PPy network. For the PPy–TSA–CNT composite (Figure 2D), the presence of carbon nanotubes is evidenced by localized linear features and punctate contrasts, which can be attributed predominantly to the exposed ends or cross-sectional projections of CNTs embedded within the PPy matrix. At the selected magnification, the nanotubes do not appear as extended continuous structures, but rather as discrete features uniformly distributed throughout the polymeric phase. This observation suggests a good dispersion of the CNTs within the PPy matrix, without the formation of large agglomerates.

The PPy–TSA–zeolite film (Figure 2E) displays a more irregular and heterogeneous surface morphology, characterized by interconnected domains and increased surface roughness. This behavior can be attributed to the inherently porous structure of the zeolite particles, which provides multiple nucleation sites and locally perturbs the growth of the PPy network during electrochemical deposition. As a result, the polymer matrix develops a more textured and heterogeneous morphology compared to the reference PPy–TSA film. The unusually high thickness of the PPy–TSA–ZE film can be attributed to the nature of the zeolite filler. The natural zeolite used in this work has a micrometric particle size (~1–5 µm) and a highly porous structure that facilitates the adsorption of pyrrole monomer, including within its microporosity. This behavior likely leads to the formation of multiple polymerization centers and promotes a more pronounced three-dimensional growth of the composite during electropolymerization. Since identical polymerization conditions and deposition time were used for all samples, the accelerated growth of the PPy–TSA–ZE system resulted in a significantly thicker film. Finally, the hybrid composite PPy–TSA–PEG–GR (Figure 2F) combines features observed in the individual additive systems, exhibiting a relatively compact polymer matrix together with embedded inorganic structures. This morphology suggests a synergistic effect of the polymeric and inorganic components on the organization of the PPy film, while preserving the characteristic PPy surface architecture. Such a morphological balance was intentionally targeted in order to enhance the mechanical stability and robustness of the films without significantly altering the intrinsic PPy microstructure.

Overall, the morphological analysis indicates that the incorporation of polymeric and inorganic additives does not induce significant detrimental changes in the structure of the PPy films. The characteristic surface morphology of electrochemically synthesized polypyrrole is maintained for all investigated compositions, while only limited and controlled modifications are observed depending on the nature of the additive. These results suggest that the structural integrity of the PPy matrix is preserved, supporting the suitability of the composite films for further functional studies.

### 3.2. Structural and Compositional Properties of PPy-Based Films

**Figure 3:**
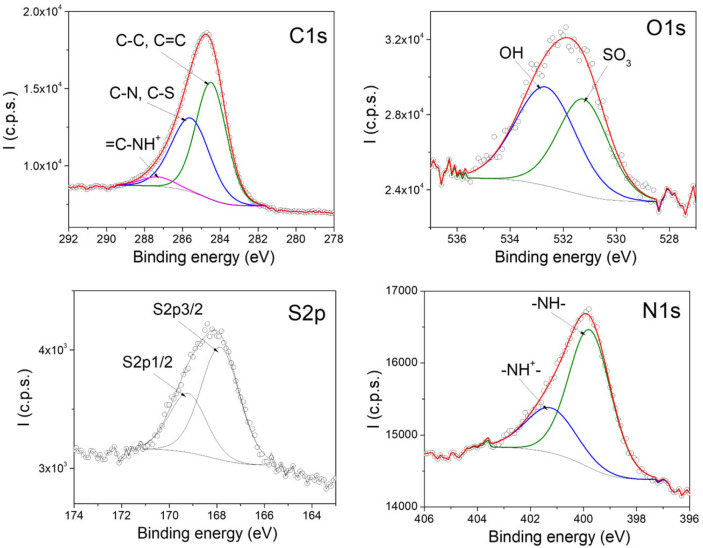
High-resolution XPS spectra of the doped PPy–TSA reference film, including the C 1s, O 1s, S 2p, and N 1s regions. (The experimental data are represented by the circle markers, while the red line corresponds to the fitted curve and the colored lines represent the individual fitting components).

X-ray photoelectron spectroscopy (XPS) was employed to investigate the surface chemical composition and doping state of the PPy films. Particular attention was paid to the N 1s core-level spectra, which provide information on the chemical environment of nitrogen atoms in the PPy backbone and on the doping state induced by p-TSA. Figure 3 presents the high-resolution XPS spectra of the doped PPy–TSA reference film, which confirms the expected surface chemical composition and doping state of electrochemically synthesized polypyrrole. The C 1s spectrum is dominated by contributions associated with the conjugated carbon framework of the PPy backbone, while the O 1s signal originates mainly from the sulfonate groups of the p-TSA dopant and from adsorbed oxygen-containing species at the film surface. The presence of sulfur clearly evidenced by the S 2p spectrum confirms the successful incorporation of p-TSA as dopant within the PPy matrix. The detection of sulfur at the film surface is consistent with protonic doping of PPy and supports the effective electrochemical polymerization process.

The N 1s core-level spectrum provides further insight into the chemical environment of nitrogen atoms in the PPy chains. The deconvolution of the N 1s peak reveals contributions corresponding to neutral amine and imine nitrogen species associated with the PPy backbone, together with a higher binding energy component attributed to positively charged nitrogen species (N^+^). The latter are commonly associated with polarons and bipolarons formed upon doping and indicate the presence of a doped conducting state in the PPy film.

Overall, the coexistence of neutral and positively charged nitrogen species, together with the presence of sulfur originating from the p-TSA dopant, confirms the successful doping of the PPy film and validates the reference material as a suitable baseline for comparison with PPy-based composite systems. These XPS results are in agreement with literature reports [34,35,36,37,38] and confirm that the chemical structure of the PPy backbone is preserved upon p-TSA doping, providing a reliable reference for evaluating the effect of polymeric, carbon-based, and inorganic additives in the composite films.

**Figure 4:**
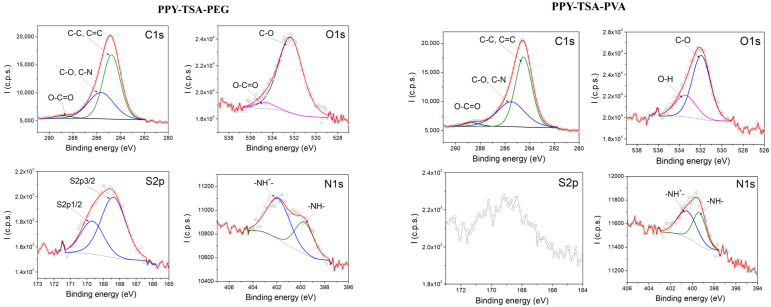
High-resolution XPS spectra of doped PPy films containing polymeric additives (P*p*y–TSA–PEG and P*p*y–TSA–PVA), including the C 1s, N 1s, O 1s, and S 2p regions. (The circle markers represent the experimental data, the red line corresponds to the overall fitted curve, and the grey line indicates the baseline, while the colored curves represent the individual fitting components).

Figure 4 shows the high-resolution XPS spectra of PPy films containing polymeric additives (PEG and PVA). In both cases, the C 1s spectra reflect the carbon-rich nature of the polymeric matrix, with relative variations in intensity associated with the presence of the polymeric additives. An increase in the O 1s contribution is observed for the PEG- and PVA-containing samples, consistent with the oxygen-containing functional groups of these polymers.

Importantly, the N 1s spectra of both composite films closely resemble that of the P*p*y–TSA reference sample, exhibiting contributions associated with neutral and positively charged nitrogen species characteristic of doped polypyrrole. The presence and shape of the S 2p signal further confirm that p-TSA doping is preserved after the incorporation of polymeric additives. Overall, these results indicate that the addition of PEG or PVA does not significantly alter the surface chemical structure or doping state of P*p*y.

**Figure 5:**
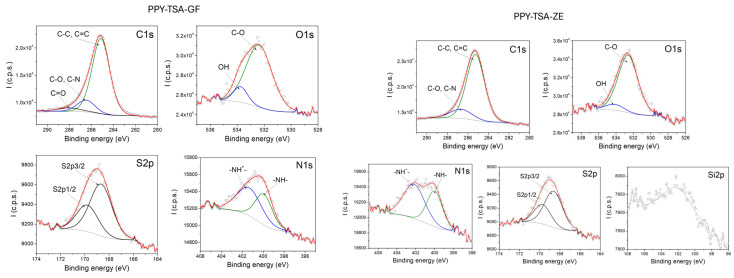
High-resolution XPS spectra of doped PPy films containing inorganic fillers (graphene-based filler and zeolite). The C 1s, N 1s, O 1s, and S 2p regions are shown. (The circle markers represent the experimental data, the red line corresponds to the overall fitted curve, and the grey line indicates the baseline, while the colored curves represent the individual fitting components).

Figure 5 presents representative high-resolution XPS spectra of doped P*p*y films containing inorganic fillers, namely graphene-based filler and zeolite. In both cases, the C 1s spectra are dominated by contributions associated with the carbon framework of the P*p*y matrix, with relative variations in intensity reflecting the carbon-rich nature of the incorporated fillers. The O 1s signal is more pronounced for the zeolite-containing film, consistent with the oxygen-rich composition and porous structure of the zeolite particles.

In addition, the XPS spectrum of the P*p*y–TSA–zeolite composite exhibits a characteristic contribution associated with the main constituent element of zeolite Si 2p, confirming the successful incorporation of the inorganic filler within the P*p*y matrix. The presence of this signal of Si 2p provides direct evidence of zeolite integration at the film surface.

Importantly, the N 1s core-level spectra of both composite films closely resemble that of the P*p*y–TSA reference sample, exhibiting contributions attributed to neutral and positively charged nitrogen species characteristic of doped polypyrrole. The presence and shape of the S 2p signal further confirm that p-TSA doping is preserved after the incorporation of inorganic fillers. No additional nitrogen- or sulfur-containing species are detected, indicating that the fundamental surface chemical structure and doping state of P*p*y remain unaffected by the presence of graphene or zeolite.

The surface atomic concentrations derived from XPS analysis for the investigated PPy-based films are summarized in Table 2. The doped PPy–TSA reference film exhibits a balanced surface composition, with carbon as the dominant element, accompanied by detectable nitrogen and sulfur contributions associated with the PPy backbone and p-TSA dopant, respectively.

A pronounced increase in the carbon atomic concentration is observed for the films containing polymeric additives (PPy–TSA–PEG and PPy–TSA–PVA), reaching values above 80 at.%. This behavior is consistent with the carbon-rich nature of PEG and PVA and indicates a significant contribution of these polymers to the surface composition. Concomitantly, a decrease in the relative nitrogen and sulfur contents is observed, which can be attributed to a partial surface coverage of the PPy matrix by the polymeric additives rather than to a loss of doping.

In the case of PPy–TSA–GR, the elevated carbon concentration reflects the incorporation of the graphene-based filler, while the presence of nitrogen and sulfur at comparable levels to the reference sample indicates that the PPy backbone and p-TSA doping are preserved. For the PPy–TSA–zeolite film, the detection of silicon, together with an increased oxygen content, provides direct evidence for the successful incorporation of the zeolite filler at the film surface.

The hybrid composite PPy–TSA–PEG–GR exhibits an intermediate surface composition, with carbon and oxygen concentrations reflecting the combined contribution of the polymeric and inorganic components, while nitrogen and sulfur remain clearly detectable. This observation suggests that the incorporation of multiple additives leads to a modified surface composition without disrupting the chemical integrity or doping state of the PPy matrix.

Overall, the XPS results indicate that the incorporation of polymeric and inorganic fillers does not induce major changes in the surface chemical structure of the PPy films.

The characteristic N 1s components associated with neutral and positively charged nitrogen species, together with the persistent S 2p signal originating from the p-TSA dopant, are maintained across all investigated compositions. While the surface atomic concentrations of C and O vary depending on the additive, no additional nitrogen- or sulfur-containing species are detected, supporting the preservation of the PPy backbone and its doping state after filler incorporation.

### 3.3. Mechanical Properties of PPy-Based Films

For bioelectronic sensing applications, such as ECG signal acquisition, the mechanical properties of electrode materials are as critical as their electrical conductivity. In flexible and skin-contact devices, the ability of a material to deform and adapt to curved or moving surfaces plays a key role in ensuring comfort, durability, and stable signal acquisition. In this context, Young’s modulus provides a straightforward indication of how a film responds to mechanical deformation: lower values correspond to softer, more compliant materials that deform easily under small stresses, while higher values indicate stiffer materials that resist deformation and may compromise conformal contact with the skin. In this work, mechanical compliance is defined as the ability of the composite films to conform to the skin surface and sustain mechanical deformation (such as bending or slight stretching) without compromising their structural integrity and electrical performance. This property is closely related to the measured Young’s modulus values discussed in this section.

To evaluate the mechanical response of the PPy-based films, dynamic mechanical analysis was performed, focusing on their elastic behavior under tensile loading. The mechanical properties were assessed from stress–strain curves, with Young’s modulus determined from the linear elastic region of the σ–ε curves. Differences in the initial slopes of these curves reflect variations in stiffness induced by film composition and provide insight into the suitability of the materials for flexible bioelectronic applications [39].

**Figure 6:**
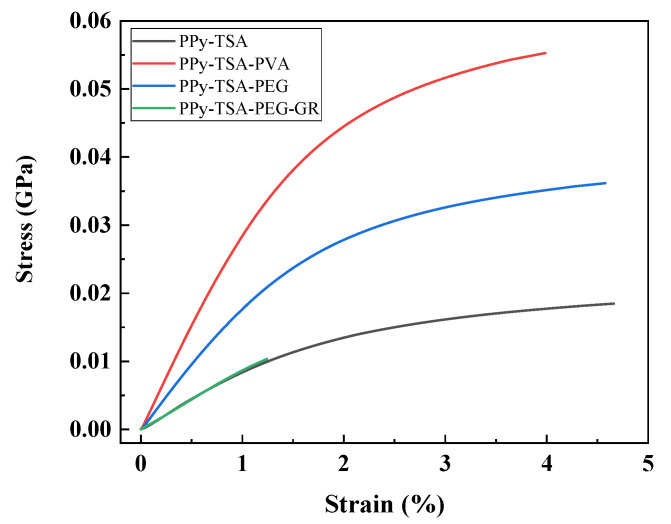
Stress–strain curves of PPy-based films without additional fillers (PPy-TSA) and with polymeric additives: PPy-TSA-PVA, PPy-TSA-PEG, and a hybrid system containing PEG and graphene (PPy-TSA-PEG-GR).

Figure 6 illustrates the influence of polymeric additives on the mechanical response of PPy-based films. The PPy film without additional fillers exhibits a low Young’s modulus of approximately 0.008 GPa, indicating a compliant material that deforms easily under low applied stresses, as commonly reported for pristine polypyrrole films [40]. While this high compliance is advantageous for flexible applications, the film shows limited mechanical resistance during handling and processing, which motivates further optimization of its mechanical properties. Overall, the mechanical response of the PPy–TSA reference film is consistent with trends reported in the literature for polypyrrole-based materials, validating its use as a suitable baseline for comparison, while the composite systems demonstrate the expected modulation of mechanical properties upon incorporation of polymeric and hybrid additives, without compromising electrical functionality [41].

The incorporation of PVA leads to a pronounced increase in stiffness, with the Young’s modulus rising to approximately 0.024 GPa. This behavior suggests that PVA acts as a reinforcing component within the PPy matrix, significantly limiting elastic deformation. In contrast, the addition of PEG results in a more moderate increase in stiffness, yielding a Young’s modulus of about 0.016 GPa. This intermediate behavior reflects the effect of PEG [42], which improves the flexibility of the film while maintaining sufficient mechanical stability, making PEG a suitable choice for further material development.

**Figure 7:**
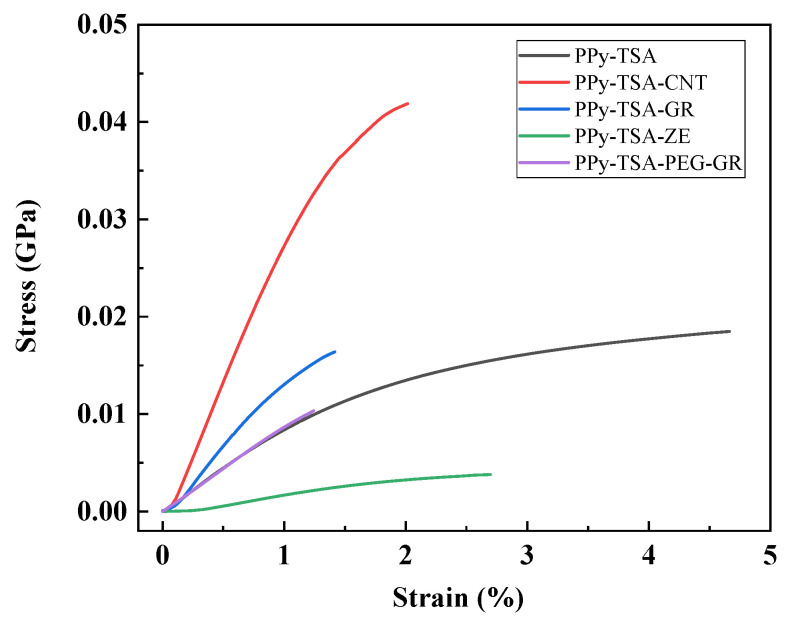
Stress–strain curves of PPy-based films without additional fillers and with inorganic fillers: carbon nanotubes (PPy-TSA-CNT), graphene (PPy-TSA-GR), zeolite (PPy-TSA-ZE), and a hybrid system containing PEG and graphene (PPy-TSA-PEG-GR).

The impact of inorganic fillers on the mechanical properties of PPy-based films is presented in Figure 7. Similar to the previous observations, the PPy film without additional fillers shows a low Young’s modulus of approximately 0.008 GPa, confirming its compliant mechanical nature [38]. The introduction of carbon nanotubes produces a strong stiffening effect, with the Young’s modulus increasing to approximately 0.026 GPa, the highest value among the investigated systems. This indicates that CNTs effectively reinforce the PPy matrix, substantially enhancing resistance to elastic deformation [43]. For the PPy–GR composite, the Young’s modulus reaches approximately 0.016 GPa, suggesting an increase in stiffness compared to the PPy film, though less pronounced than in the case of CNT incorporation. By contrast, the PPy–ZE film exhibits a very low Young’s modulus of approximately 0.003 GPa, indicating a highly compliant material with minimal resistance to deformation at low stresses. Among the inorganic fillers investigated, graphene was selected as the most suitable additive, as it does not significantly alter the elastic behavior of the initial PPy film, while providing improved mechanical robustness and facilitating easier handling and processing of the material [44]. The decrease in Young’s modulus observed for the zeolite-containing films may appear counterintuitive, as rigid inorganic fillers are typically expected to increase the stiffness of polymer composites. However, this behavior can be explained by several structural factors. Zeolites are highly microporous materials, which can introduce additional free volume and structural heterogeneity into the polymer matrix, promoting the formation of microvoids. In addition, weak interfacial interactions between PPy and zeolite particles may lead to inefficient stress transfer and local debonding under mechanical load. Similar effects have been reported in polymer composites, where poor interfacial adhesion and filler agglomeration reduce the reinforcing efficiency of rigid fillers and may even decrease stiffness [45,46,47].

Furthermore, the zeolite-containing films exhibited a higher thickness compared to the neat PPy films, suggesting a less compact structure. Increased thickness, particularly in systems containing porous fillers, can be associated with a higher degree of structural heterogeneity and reduced packing density, which further contributes to the observed reduction in Young’s modulus.

Considering the positive influence of PEG observed among the polymeric additives, as well as the favorable effect of graphene identified among the inorganic fillers, a composite system containing both components was further investigated. The mechanical response of the PPy–PEG–graphene composite reflects the combined influence of a polymeric modifier and a conductive inorganic filler. The Young’s modulus of this system is approximately 0.009 GPa, close to that of the PPy film without additives, indicating that the presence of PEG counterbalances the stiffening effect typically induced by graphene.

As a result, the PPy–PEG–GR composite maintains a compliant mechanical behavior while benefiting from composite formation, highlighting a favorable balance between flexibility and structural reinforcement. This combination is particularly relevant for applications requiring both mechanical adaptability and functional performance, such as flexible bioelectronic electrodes.

From this perspective, the mechanical analysis reveals clear differences among the investigated PPy-based films. Systems exhibiting high Young’s modulus values behave as relatively rigid materials, which may limit their ability to conform to the skin and maintain stable contact during ECG monitoring. In contrast, films with low Young’s modulus values display enhanced mechanical compliance, allowing easier deformation and improved adaptability to skin movements. Among the studied compositions, the PPy–PEG–GR composite achieves this balance most effectively, maintaining a low elastic modulus comparable to that of the PPy film without additives, while simultaneously benefiting from the functional advantages introduced by composite formation. This makes it particularly well-suited for use as a flexible, gel-free ECG electrode.

### 3.4. Electrical Properties of PPy-Based Films

In the first stage, a comparative analysis of the electrical conductivities of the different films was carried out at room temperature (25 °C) in order to assess the influence of the various additives and film thickness on charge transport properties. The reported conductivity values were calculated by considering the specific geometry of the samples, including film thickness and probe configuration, and by applying the appropriate geometrical correction factors.

The room-temperature conductivity values obtained for all samples are summarized in Table 3. For comparison purposes, representative conductivity ranges reported in the literature for similar PPy-based systems are included.

The experimental values obtained in this study fall within the expected intervals, confirming the reliability of the measurements and the consistency of the observed trends. The reference PPy–TSA film exhibits a conductivity of 208 S/cm, which is in very good agreement with literature data reported for electrochemically synthesized polypyrrole doped with p-toluenesulfonic acid, where conductivities typically range from tens to several hundred S/cm, depending on the synthesis conditions, dopant concentration, and film morphology. This result confirms the effective doping of the PPy backbone and the formation of a well-connected conjugated network.

The incorporation of insulating polymeric additives leads to a noticeable decrease in conductivity. In the case of PPy–TSA–PEG, the conductivity drops to 38 S/cm. This behavior is commonly attributed to the insulating nature of polyethylene glycol, which dilutes the conducting phase and increases the average distance between conjugated PPy chains, thereby reducing charge carrier mobility. Similar reductions in conductivity have been widely reported for PPy/PEG systems, particularly at PEG contents of around 1 wt%.

A less pronounced decrease is observed for the PPy–TSA–PVA sample, which shows a conductivity of 101 S/cm. Compared to PEG, poly (vinyl alcohol) can establish hydrogen bonding interactions with the PPy matrix, which may partially preserve interchain connectivity and result in a moderate rather than drastic reduction in conductivity.

The addition of carbon-based conductive fillers at low loadings (0.15 wt%) does not lead to an enhancement of electrical conductivity. For the PPy–TSA–GR sample, a conductivity of 62 S/cm is obtained, while the PPy–TSA–CNT film exhibits similar value of 58 S/cm. Although graphene and carbon nanotubes are intrinsically highly conductive, their effectiveness strongly depends on the formation of a percolating network within the polymer matrix. At the relatively low filler concentrations used in this study, the percolation threshold is not reached, and the fillers may instead disrupt the electrochemical growth and continuity of the PPy phase. Similar trends have been reported in the literature for PPy/GR and PPy/CNT composites below the percolation threshold, where conductivity either remains unchanged or decreases relative to pristine PPy.

A pronounced reduction in conductivity is observed for the PPy–TSA–ZE sample, which exhibits a value of 4.5 S/cm. This behavior can be attributed to the insulating nature of zeolite, combined with the significantly increased film thickness (~150 µm) compared to the other samples. The incorporation of zeolite likely introduces a highly heterogeneous and porous structure, leading to disrupted conductive pathways and increased bulk resistance. Comparable conductivity values have been reported for PPy composites containing inorganic insulating fillers such as silica, aluminosilicates, or zeolites.

The ternary PPy–TSA-PEG–GR system shows an intermediate conductivity of 43 S/cm, reflecting the combined influence of an insulating polymer (PEG) and a conductive carbon filler (graphene). While PEG reduces the overall conductivity by diluting the PPy network, the presence of graphene partially compensates for this effect by improving local charge transport pathways, even though a fully percolating graphene network is not formed. Similar intermediate conductivities have been reported for ternary PPy-based hybrid systems, highlighting the balance between processability, mechanical modification, and electrical performance.

Overall, the conductivity data measured at ambient temperature (25 °C) demonstrate that the fundamental conduction mechanism of p-TSA-doped polypyrrole is preserved across all composite systems, while the absolute conductivity values are influenced by the nature of the additives, which in turn affects the resulting film thickness. The conductivity values obtained are within the range typically reported for materials considered suitable for electrocardiographic (ECG) sensing and related bioelectronic applications, while simultaneously allowing an improved balance between electrical conductivity and mechanical performance. These features make the prepared PPy-based composite films promising candidates for flexible bioelectronic interfaces.

**Figure 8:**
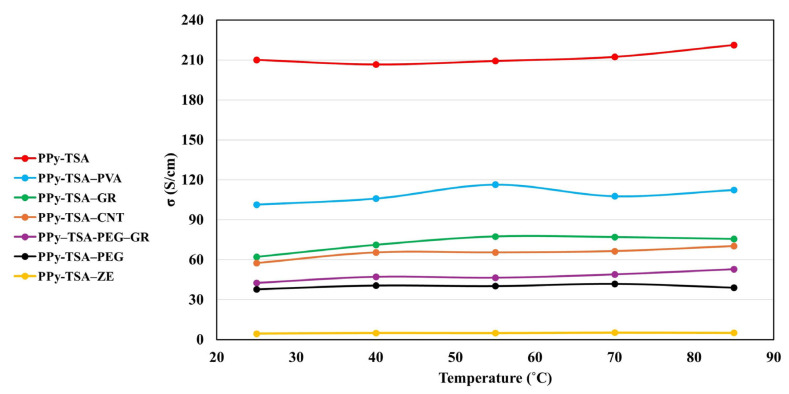
Temperature-dependent electrical conductivity of pristine PPy–TSA and composite PPy-based films (PPy–TSA–PVA, PPy–TSA–PEG, PPy–TSA–GR, PPy–TSA–CNT, and PPy–TSA–ZE) measured in the range of 25–85 °C.

To further evaluate the suitability of the PPy-based films for biomedical sensing applications, the electrical conductivity was investigated as a function of temperature in the range of 25–85 °C. This interval was selected to assess the thermal stability of the electrical response under conditions relevant to wearable devices, where moderate temperature variations may occur due to body heat or environmental temperature variation. As shown in Figure 8, all samples exhibit relatively stable conductivity values over the investigated temperature range, with only moderate variations. The pristine PPy–TSA film displays a slight increase in conductivity with increasing temperature, which is characteristic of thermally activated charge transport in doped conducting polymers. Such behavior is typically associated with a hopping conduction mechanism occurring along and between polymer chains.

The incorporation of secondary fillers (PVA, GR, CNT, PEG, and ZE) does not induce thermal instability. In most composite systems, the conductivity either increases slightly or remains nearly constant with temperature, indicating preserved charge transport pathways and stable interfacial interactions within the hybrid structure.

In agreement with literature reports on doped polypyrrole systems—where conductivity generally increases moderately with temperature due to enhanced carrier mobility and hopping probability—the present results confirm the expected semiconducting behavior while demonstrating improved thermal stability in the composite films. Importantly, no abrupt transitions or conductivity degradation were observed, suggesting that the materials maintain reliable electrical performance within the investigated temperature range.

Overall, the results confirm that the incorporation of secondary fillers allows for fine-tuning of the electrical properties without compromising the intrinsic conduction mechanism or thermal stability of the PPy–TSA matrix.

### 3.5. Optical Properties of PPy-Based Thin Films

**Figure 9:**
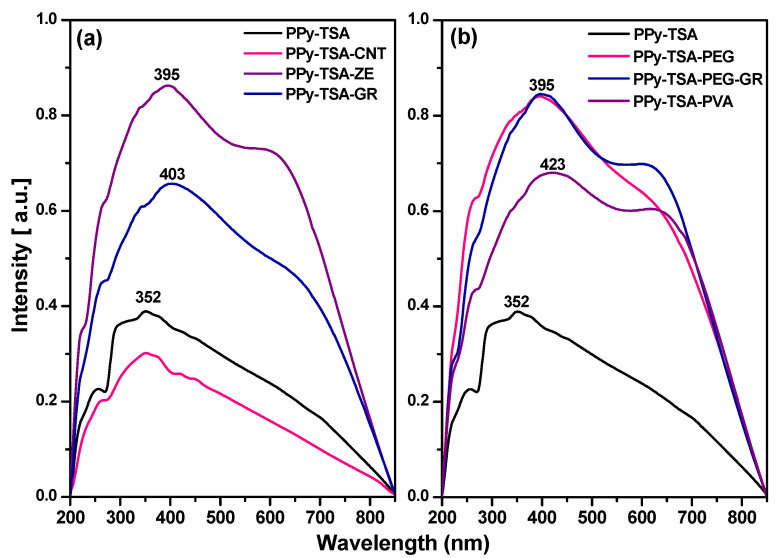
UV–Vis absorption spectra of TSA-doped polypyrrole reference (PPy-TSA) and the corresponding composite films: (**a**) composites containing carbon-based and inorganic fillers (PPy-TSA-CNT, PPy-TSA-ZE, PPy-TSA-GR); (**b**) composites containing polymeric and hybrid fillers (PPy-TSA-PEG, PPy-TSA-PEG-GR, PPy-TSA-PVA).

The UV–Vis spectra of the TSA-doped PPy reference and the corresponding composite films are presented in Figure 9. All samples exhibit broad absorption features characteristic of doped π-conjugated polymers [63,64,65]. The absorption observed below 300 nm is associated with short conjugated segments and benzenoid structures of the PPy backbone, with possible contributions from the aromatic rings of the incorporated TSA dopant [66,67,68]. The band located in the 300–350 nm region corresponds to the fundamental π–π* transition of the PPy backbone and is highly sensitive to the oxidation level and effective π-electron delocalization along the conjugated chain

For the carbon-based and inorganic composites (Figure 9a), distinct spectral differences are observed compared to the PPy-TSA reference. In the CNT-containing samples, the π–π* maximum remains centered around ~352 nm, similar to the reference, and the VIS–NIR absorption band exhibits only moderate enhancement. These features indicate that the intrinsic electronic structure of the PPy backbone is only weakly affected by CNT incorporation. Although CNTs are intrinsically conductive, the absence of a significant redshift and the limited VIS–NIR enhancement suggest that effective electronic coupling and percolation within the composite remain limited. This behavior is consistent with the observed reduction in macroscopic electrical conductivity.

In contrast, the graphene- and zeolite-containing samples show a clear redshift of the π–π* transition toward ~397 nm, accompanied by an enhanced absorption band in the 550–680 nm region. The redshift indicates increased effective conjugation length and modified electronic structure of the PPy matrix. For graphene-containing films, π–π interactions between graphene sheets and PPy chains may promote local delocalization of charge carriers. However, despite these optical signatures, the electrical conductivity decreases, suggesting that long-range charge transport is hindered by incomplete conductive pathways or interfacial charge-transfer barriers. In the case of zeolite-containing films, although enhanced VIS–NIR absorption indicates increased polaron/bipolaron formation, the insulating nature of the zeolite disrupts the continuity of the conductive network, leading to a pronounced decrease in conductivity.

A different behavior is observed for the polymer-containing composites (Figure 9b). The incorporation of PEG- and PVA-based fillers induces a pronounced redshift of the π–π* transition, from ~352 nm for PPy-TSA to ~397 nm for PEG-containing films and up to ~426 nm for PVA-containing films. This shift suggests increased effective conjugation length and enhanced π-electron delocalization. In addition, a strong and broadened absorption band in the 550–680 nm region extending toward the NIR domain is observed, characteristic of bipolaronic transitions in highly doped PPy. The intensified VIS–NIR absorption indicates an increased density of charge carriers, likely facilitated by improved chain packing and stabilization of the doped state through polymer–polymer interactions.

Among these samples, the PVA-containing composite exhibits the most pronounced redshift and VIS–NIR enhancement, correlating with its higher conductivity compared to the PEG-containing films. Nevertheless, its conductivity remains lower than that of the PPy-TSA reference, which can be attributed to partial dilution of the conductive phase and disruption of continuous charge-transport pathways by the insulating polymer matrix.

Overall, the UV–Vis analysis demonstrates that the incorporation of different fillers alters the electronic structure of the PPy matrix, as evidenced by systematic shifts in the π–π* transition and variations in the VIS–NIR absorption intensity. Polymeric additives induce the most pronounced redshift, indicating enhanced effective conjugation and increased charge-carrier density, whereas carbon-based and inorganic fillers produce more composition-dependent modifications. Importantly, the optical behavior does not directly translate into proportional changes in electrical conductivity. This indicates that, beyond local electronic structure, macroscopic charge transport in these composites is strongly governed by phase continuity, interfacial interactions, and percolation pathways within the film.

**Figure 10:**
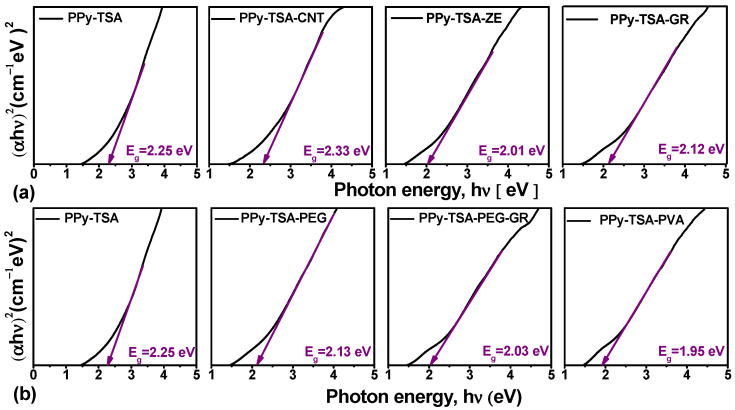
Tauc plots $(\alpha h\nu {)}^{2}$ versus photon energy for the reference PPy-TSA film and the corresponding composite films: (**a**) Samples doped with inorganic fillers; (**b**) Samples containing polymeric additives.

The optical band gap (Eg) of the reference film PPy-TSA and the corresponding composite films were estimated from the UV–Vis absorption spectra using the Tauc relation:

$$\alpha h\nu ={A\;(h\nu -E_{g})}^{n}$$

where α is the absorption coefficient, hν is the photon energy (eV), A is a constant, and *n* = 2 corresponds to indirect allowed transitions. The Eg values were determined from the intercept of the linear region of the (*αhν*)^2^ versus photon energy plots (Figure 10).

In its neutral state, polypyrrole (PPy) behaves as an electrical insulator with a relatively high band gap, typically reported in the range of 3.16–3.46 eV [69,70]. However, due to its conjugated backbone of alternating single and double bonds, PPy becomes electrically conductive upon chemical oxidation (doping). During oxidation, π-electrons are partially removed from the polymer chain, leading to the formation of polarons and bipolarons. These charged species introduce new electronic states within the forbidden energy gap and promote a structural transition from benzenoid to a more quinoid-like configuration, enabling doped PPy to function as a semiconductor.

The estimated optical band gap of the reference PPy-TSA film is 2.25 eV, confirming its doped semiconducting character. For the carbon-based composites, PPy-TSA-CNT exhibits a slight increase in Eg to 2.33 eV. This increase suggests that the incorporation of CNTs may partially disturb the effective conjugation length of the PPy backbone or introduce localized structural disorder, thereby limiting π-electron delocalization.

In contrast, the incorporation of polymeric and hybrid fillers leads to a progressive reduction in the optical band gap. Specifically, PPy-TSA-PEG shows a decrease compared to PPy-TSA, followed by PPy-TSA-GR and PPy-TSA-PEG-GR, which exhibit intermediate Eg values. A more pronounced reduction is observed for PP-TSA-ZE, while the lowest Eg value (1.95 eV) is obtained for PP-TSA-PVA.

The reduction in Eg for these composites is consistent with enhanced π-electron delocalization and an increased density of polaronic and bipolaronic states, as also supported by the redshift of the π–π* transition and the intensified VIS–NIR absorption bands observed in the UV–Vis spectra. The formation of these intermediate electronic states effectively narrows the optical band gap.

Overall, the band gap values highlight the influence of the filler nature on the electronic structure of the PPy matrix, while remaining within a relatively narrow range. This behavior is particularly advantageous, as the fillers were primarily introduced to enhance mechanical performance, and the results demonstrate that these modifications do not compromise the fundamental semiconducting properties of the material.

### 3.6. Application of PPy-Based Conductive Films for ECG Signal Recording

Beyond structural and electrical characterization, the practical applicability of the PPy-based conductive films was assessed through their use as active electrodes for electrocardiographic (ECG) signal acquisition. Owing to their intrinsic electrical conductivity, mechanical compliance, and good adhesion, the developed PPy films are promising candidates for skin-contact bioelectronic interfaces.

The PPy-based ECG electrode was fabricated by attaching a thin silver wire onto the surface of the conductive PPy film to ensure reliable electrical contact. The resulting assembly was subsequently fixed onto a soft, skin-friendly insulating polymer support, yielding a flexible electrode suitable for direct skin contact and ECG signal acquisition. This straightforward electrode configuration highlights the ease of integrating PPy-based films into functional ECG sensors without the need for complex processing steps. Moreover, the developed electrode operates in a gel-free configuration and exhibits good adhesion to the skin, maintaining stable contact under dynamic conditions and in the presence of perspiration or moisture.

In addition, the compact size of the active PPy film highlights the potential for further miniaturization of the electrode, suggesting that future optimization could enable even more compact device designs without compromising functionality.

**Figure 11:**
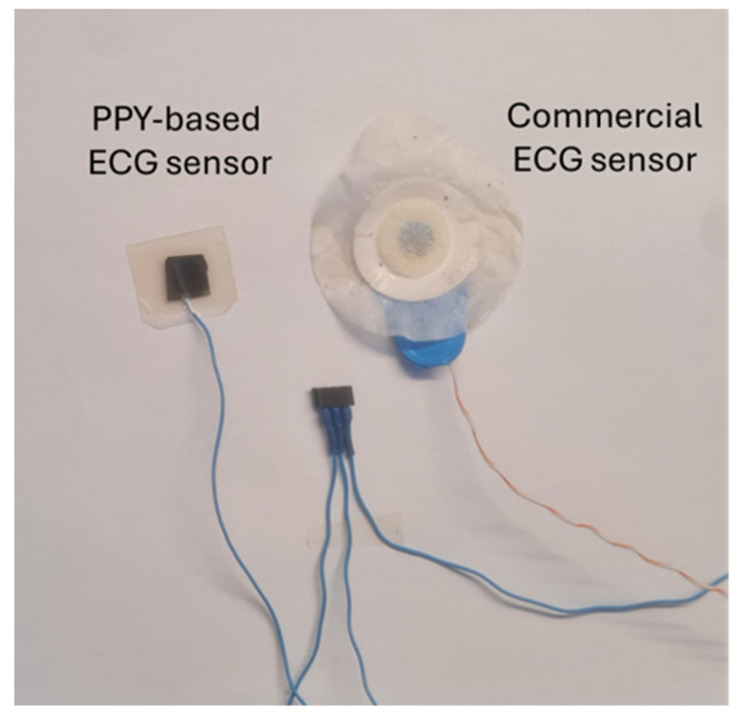
Comparison between a commercial ECG electrode and a PPy-based ECG electrode, illustrating the simplicity and miniaturization potential of the PPy-based electrode design.

A direct visual comparison between a commercial ECG electrode and the PPy-based electrode developed in this work is presented in Figure 11, highlighting the practical implementation and compact design of the PPy-based sensing platform.

The ECG recordings were performed to evaluate the ability of the PPy-based electrodes to reliably capture ECG signals and to compare their performance with that of a commercial ECG electrode. To ensure a reliable comparison, both the commercial ECG electrode and the PPy-based electrodes were positioned in close proximity, enabling signal acquisition from the same skin area.

**Figure 12:**
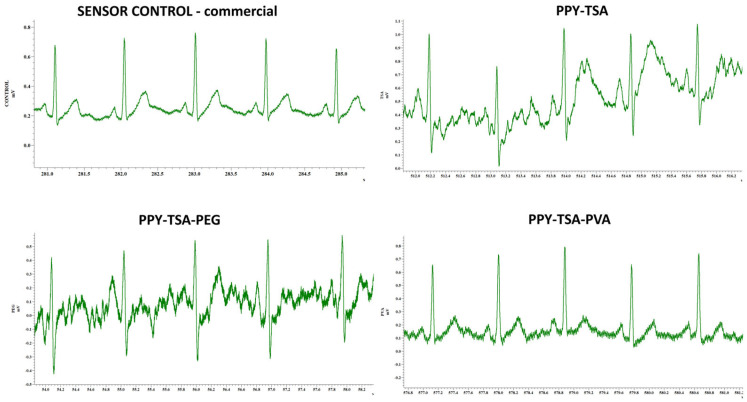

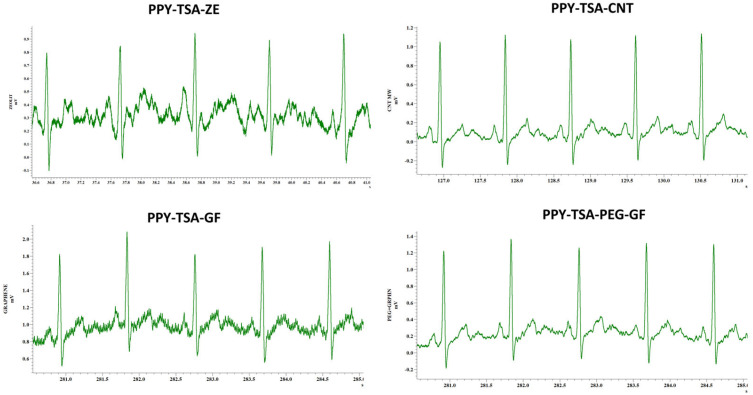
Representative ECG signals recorded using a commercial control sensor and PPy-based electrodes with different compositions (PPy–TSA, PPy–TSA–PEG, PPy–TSA–PVA, PPy–TSA–ZE, PPy–TSA–CNT, PPy–TSA–GR, and PPy–TSA–PEG–GR), acquired under identical experimental conditions.

Representative ECG signals recorded using PPy-based film electrodes with different compositions, together with those recorded using a commercial control ECG electrode, are presented in Figure 12. As already mentioned in Section 2.4, all ECG recordings were performed on a subject under resting conditions using identical recording settings, which thus allows a qualitative comparison of the ECG signal morphology obtained with the different PPy-based electrodes and the commercial ECG electrode. This analysis aims to assess the influence of the PPy film composition on the ability of the conductive polymer electrodes to capture biopotentials characterized by high stability and signal-to-noise ratio.

As shown in Figure 12, the ECG signals recorded with the commercial electrode exhibit the characteristic P wave, QRS complex, and T wave components, thus serving as reference for the morphology and temporal features of the ECG signals recorded with the PPy electrodes. The fact that the same ECG components can be identified in the ECG signals recorded using all PPy-based electrodes indicates that the conductive polymer films tested in this study were capable of detecting cardiac electrical activity regardless of their composition.

The TSA-doped PPy electrode provided clear ECG signals with well-defined QRS complexes, although the baseline fluctuations that are observed in the recording may indicate skin–electrode contact variations in the absence of a conductive gel.

The incorporation of the polymeric additive PEG and PVA leads to ECG instability in the first case and noise contamination in the second. However, the ECG features remained clearly distinguishable in the recordings, with PPy-TSA-PVA electrodes confirming that these films retain sufficient electrical conductivity for ECG signal acquisition.

The addition of conductive fillers, including carbon nanotubes and graphene, results in improved signal stability and better signal-to-noise, suggesting more efficient charge transport within the composite films. In particular, the PPy–graphene PPy–TSA-CNT and PPY-TSA-PEG-GR electrodes exhibited more stable, well-defined and noise-free ECG components compared to the polymer-modified systems.

The combined PPy–PEG–graphene films benefit from both the mechanical compliance introduced by PEG and the enhanced electrical pathways provided by graphene, leading to ECG signals with improved waveform definition relative to PPy–PEG alone.

Overall, the ECG recordings demonstrate that PPy-based conductive films with different compositions can function as electrodes for ECG signal acquisition. The observed differences in signal stability, morphology and noise contamination highlight the role of film composition in tuning the electrochemical and interfacial properties of the electrodes, while confirming the feasibility of PPy-based materials for gel-free bioelectronic sensing applications. Among the investigated systems, the PPy–PEG–graphene composite film simultaneously fulfills the key requirements for ECG signal acquisition, combining adequate electrical conductivity, mechanical compliance, and stable signal transduction at the skin–electrode interface. This conclusion is fully consistent with the structural, morphological, and electrical characterization results discussed in the previous sections.

In the context of previously reported flexible electrode materials, conductive polymers such as polypyrrole (PPy) and PEDOT: PSS, as well as carbon-based systems (graphene, carbon nanotubes), have been extensively investigated for biomedical sensing applications. While pristine PPy exhibits good electrical conductivity, it often suffers from limited mechanical strength and flexibility, which may affect long-term stability and conformal skin contact. To overcome these limitations, composite strategies have been developed to improve the mechanical robustness and elasticity of the material. Compared to purely polymeric systems, the incorporation of inorganic fillers provides additional mechanical reinforcement without significantly compromising conductivity. In line with these reports, the PPy-based composite films developed in this work combine good electrical performance with improved mechanical properties, enabling stable ECG signal acquisition comparable to commercial electrodes.

The obtained results demonstrate that the developed PPy-based composite films combine suitable electrical conductivity, improved mechanical properties, and stable ECG signal acquisition. These characteristics highlight their strong potential for wearable ECG sensing applications, particularly for flexible, gel-free bioelectronic devices.

## 4. Conclusions

In this study, flexible p-TSA-doped polypyrrole (PPy–TSA) composite films were successfully synthesized via electrochemical polymerization and systematically modified using polymeric (PEG, PVA) and inorganic fillers (graphene, CNTs, zeolite), as well as their hybrid combinations in order to tailor the mechanical and electrical properties of the materials for electrocardiographic (ECG) sensing applications.

Morphological analysis confirmed that the incorporation of additives induced controlled modifications of the PPy surface without disrupting the characteristic cauliflower-like microstructure of electrochemically synthesized polypyrrole. XPS investigations demonstrated that the chemical structure and doping state of the PPy backbone were preserved across all composite systems, confirming the stability of the p-TSA-doped conducting state after filler incorporation.

Mechanical characterization revealed significant modulation of Young’s modulus depending on additive type. Polymeric and carbon-based fillers increased stiffness to different extents, while the hybrid PPy–PEG–graphene system maintained a low elastic modulus (~0.009 GPa), comparable to pristine PPy, thus ensuring mechanical compliance suitable for skin-contact applications, enabling conformal contact with the skin and accommodation of mechanical deformation.

Electrical measurements showed that all composite films retained conductivities within the range required for ECG sensing, despite composition-dependent reductions relative to pristine PPy–TSA. Temperature-dependent studies (25–85 °C) confirmed stable semiconducting behavior and preserved charge transport mechanisms in all systems. Optical characterization further indicated that additive incorporation modifies π–π* transitions and polaron/bipolaron absorption features, although macroscopic conductivity remained governed by percolation and phase continuity effects.

Practical validation through ECG signal recording demonstrated that all PPy-based films synthetized in this study are capable of detecting cardiac biopotentials in a gel-free configuration. Among the investigated systems, the PPy–PEG–graphene composite exhibited the most favorable balance between mechanical flexibility, electrical performance, and signal stability, providing ECG waveforms comparable to those obtained with a commercial ECG electrode.

Overall, the results demonstrate that controlled composite engineering of doped polypyrrole enables the development of flexible, gel-free bioelectronic electrodes with tunable mechanical and functional properties, highlighting the strong potential of PPy-based composite films for wearable ECG sensing applications.

## Figures and Tables

**Table 1:** Synthesis parameters for PPy–TSA reference and PPy-based composite films.

Sample Name	Py Conc (M)	p-TSA Conc. (M)	Additive	Additive Conc. (wt%)	Film Thickness (µm) *
PPy-TSA	0.1 M	0.1 M	-	-	22.8 ± 1.3
PPy-TSA–PEG	0.1 M	0.1 M	PEG	18	22.3 ± 1.6
PPy-TSA–PVA	0.1 M	0.1 M	PVA	13	19.18 ± 0.9
PPy-TSA–GR	0.1 M	0.1 M	GR	0.4	22.38 ± 1.1
PPy-TSA–CNT	0.1 M	0.1 M	CNT	0.4	25.62 ± 1.9
PPy-TSA–ZE	0.1 M	0.1 M	ZE	4	152.3 ± 11.4
PPy–TSA-PEG–GR	0.1 M	0.1 M	PEG + GR	PEG: 18 GR: 0.4	15.98 ± 0.8

* Thickness values obtained from cross-sectional SEM images (average ± standard deviation).

**Table 2:** Surface atomic concentrations (at. %) of doped PPy and PPy-based composite films determined by XPS.

Sample	Atomic Concentration (%)				
	C	O	N	S	Si
Ppy-TSA	64.03	21.74	6.70	7.53	-
PPy-TSA-PEG	81.65	14.70	1.80	1.85	-
PPy-TSA-PVA	82.23	15.75	1.28	0.74	-
PPy-TSA-GR	80.59	14.65	2.29	2.47	-
PPy-TSA-ZE	77.09	16.55	2.95	2.38	1.03
PPy-TSA-PEG-GR	72.35	20.45	5	2.2	-

**Table 3:** Electrical conductivity of PPy-based films measured at room temperature (25 °C).

Sample Name	Electrical Conductivity (S·cm^−1^) at 25 °C	Literature Values (S·cm^−1^)	Ref.
PPy–TSA	208 ± 16	50–300	[48,49]
PPy–TSA–PEG	38 ± 3	10–80	[50,51]
PPy–TSA–PVA	101 ± 14	10^−3^–10	[52,53,54]
PPy–TSA–GR	62 ± 3	2–100	[55,56]
PPy–TSA–CNT	58 ± 8	2–10	[57,58,59]
PPy–TSA–ZE	4.5 ± 0.6	10^−6^–10	[60,61,62]
PPy–TSA-PEG–GR	43 ± 4	-	-

## Data Availability

The data presented in this study are available on request from the corresponding author.
